# The High Origin of the Radial Artery (Brachioradial Artery): Its Anatomical Variations, Clinical Significance, and Contribution to the Blood Supply of the Hand

**DOI:** 10.1155/2018/1520929

**Published:** 2018-06-11

**Authors:** Robert Haładaj, Grzegorz Wysiadecki, Zbigniew Dudkiewicz, Michał Polguj, Mirosław Topol

**Affiliations:** ^1^Department of Normal and Clinical Anatomy, Interfaculty Chair of Anatomy and Histology, Medical University of Lodz, Poland; ^2^Clinic of Hand Surgery, Chair of Traumatology and Orthopaedics, Medical University of Lodz, Poland; ^3^Department of Angiology, Interfaculty Chair of Anatomy and Histology, Medical University of Lodz, Poland

## Abstract

**Background:**

This study thoroughly analyzes the anatomic variations of the brachioradial artery (radial artery of high origin) based on the variability of its origin, the presence and types of anastomosis with the brachial artery in the cubital fossa (“cubital crossover” or “cubital connection”), and the pattern of radial recurrent arteries, as well as the vascular territory within the hand.

**Material and Methods:**

One hundred and twenty randomly selected, isolated upper limbs fixed in 10% formalin solution were dissected.

**Results:**

The radial artery was found to have a high origin in 9.2% of total number of the limbs: two cases from the axillary artery; nine cases from the brachial artery. Anastomosis between the brachioradial and “normal” brachial arteries in the cubital fossa was also frequently observed (54.6%). The anastomosis (“cubital crossover”) was dominant in one case, balanced in three cases, minimal in two cases, and absent in five cases.

**Conclusions:**

The brachioradial artery may originate from the brachial and, less frequently, from the axillary artery. Anastomosis between the brachioradial and “normal” brachial arteries in the cubital fossa may be dominant, balanced, minimal, or absent. A complete radioulnar arch was found more often when the brachioradial artery was present as a variant.

## 1. Background

A comprehensive understanding of the possible arrangements of the arterial pattern of the upper limb is of great clinical importance [1–6]. In particular, the radial artery demonstrates high anatomical variability regarding its origin [7–15], various arrangements of radial recurrent arteries [16–18], and the vascular territory within the hand [6, 10, 19]. The origin of the radial artery is commonly located in the cubital fossa at the level of the neck of the radius [20]. However, the artery can display a high origin from the brachial artery or even from the axillary artery [7, 8, 10, 12–14, 21, 22]. Moreover, in rare cases, the radial artery may demonstrate a more distal origin, under the pronator teres muscle [15, 23–25], or even it can be absent [26].

Various terminology has been used to describe the high origin of the brachial artery, for example, a radial artery originating from the brachial (or axillary) artery [10], a high bifurcation of the brachial artery [27], the continuance of the superficial brachial artery as the radial artery [28, 29], or a double brachial artery [30]. Rodríguez-Niedenführ et al. [12, 13] propose a clear and unified nomenclature for these variations, with the term “brachioradial artery” being used for the “high origin of the radial artery”. In the case of the brachioradial artery, the accessory artery is observed in the medial bicipital sulcus, running superficially to the median nerve and continuing along the forearm as the radial artery, following its usual course; in this variant, the brachial artery assumes a typical position and runs deep to the median nerve. The brachioradial artery often forms an anastomosis with the “normal” brachial artery in the cubital fossa (so-called “cubital crossover” or “cubital connection”) [10, 12, 13, 17].

Since the radial artery is often used in vascular, plastic, and reconstructive surgery [2, 3, 31], as well as for arterial puncture and cannulation (transradial access) [32–35], knowledge of its variations can be of great clinical significance. In this context, it is also important to know the possible anatomic variations of the brachioradial artery, including the variability of its origin, the presence of anastomosis with the brachial artery in the cubital fossa (“cubital crossover”), the pattern of recurrent radial arteries, and the vascular territory in the hand. This is the study summarizing current knowledge of the anatomy of the brachioradial artery, based on all the variables listed above. The study also sets out a proposal of unified classification of the “cubital crossover” and discusses the developmental background of the observed variations.

## 2. Material and Methods

One hundred and twenty randomly selected, isolated upper limbs fixed in 10% formalin solution were dissected. The limbs came from male cadavers (total 65, right 36, left 29) and from female cadavers (total 55, right 27, left 28). The study was approved by the Local Ethics Committee (No: RNN/517/14/KB).

The research was carried out in accordance with anatomical dissection techniques. Prior to each procedure, a thorough visual external inspection was performed to exclude specimens with deformations or traces of trauma or surgical procedures. Furthermore, limbs demonstrating anatomical variations of the arterial pattern not associated with the radial or brachioradial arteries and their branches were also excluded from further analysis. The classification of anatomical variations of the arterial pattern in the upper limb used in this study was based on those proposed by Rodríguez-Niedenführ et al. [12, 13, 17]. The preparation of blood vessels was performed using microsurgical instruments at a magnification of 2.5 x; these procedures were performed using HEINE® HR 2.5 X High Resolution Binocular Loupe (HEINE Optotechnik GmbH & Co. KG, Herrsching, Germany).

A Digimatic Caliper (Mitutoyo Corporation, Kawasaki-shi, Kanagawa, Japan) was used to take the following measurements: vessel diameter, the distance between the origin of the radial or brachioradial artery, and the intercondylar line of the humerus. Each measurement was taken twice, and the mean of both measurements was accepted as the final result. The Chi Square test was used to test relationships between selected variables, for example, the frequency of occurrence of the brachioradial artery with regard to sex or side of the body. The significance level adopted in the analysis was p = 0.05. The calculations were made with STATISTICA software, version 10.0 PL.

## 3. Results

### 3.1. Frequency of the High Origin of the Radial Artery (Brachioradial Artery)

Among the 120 examined upper limbs, the radial artery was found to have a high origin in 11 specimens (11/120; 9.2% of the total number of limbs): six male limbs (6/65; 9.2% of male limbs) and five female limbs (5/55; 9.1% of female limbs). Moreover, this variation was found on the right side in six cases (6/63; 9.5% of right limbs) and on the left side in five cases (5/57; 8.8% of left limbs). No statistically significant difference was found between both the frequency of the occurrence of the brachioradial artery and either sex or the side of the body.

### 3.2. Anatomical Variations of the Origin of the Brachioradial Artery

**Figure 1:**
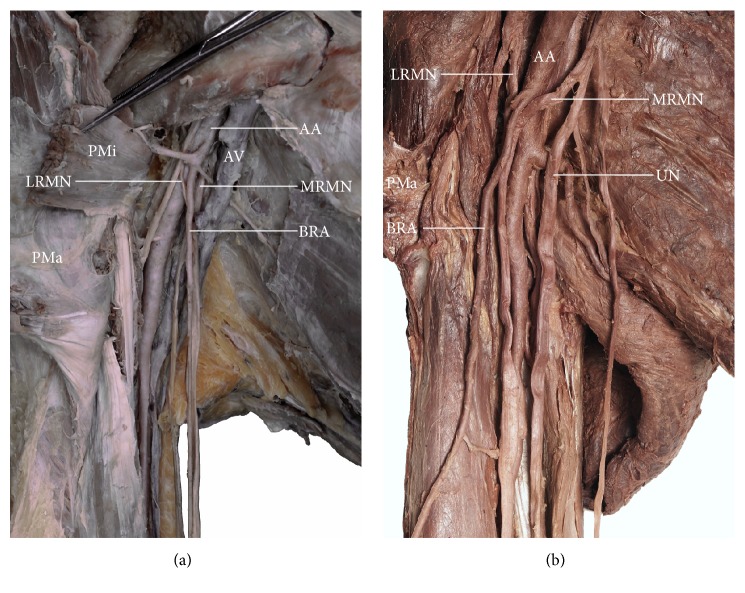
Two variants of the brachioradial artery originating within the axillary fossa. In both cases the brachioradial artery ran anterior to the roots of the median nerve. (a) Brachioradial artery arising from the first part of the axillary artery, just above the medial border of the pectoralis minor muscle. (b) Brachioradial artery branching off the second part of the axillary artery (posterior to the pectoralis minor muscle).* AA*: axillary artery;* AV*: axillary vein;* BRA*: brachioradial artery;* LRMN*: lateral root of the median nerve;* MRMN*: medial root of the median nerve;* PMa*: pectoralis major muscle;* PMi*: pectoralis minor muscle;* UN*: ulnar nerve.

The brachioradial artery was found to arise in the axillary cavity in two out of 120 dissected upper limbs (i.e., 1.67% of all limbs and 18.1% of brachioradial arteries); both cases were observed in male, right limbs. In one specimen, a thin and hypoplastic brachioradial artery branched off the first part of the axillary artery, just above the medial border of the pectoralis minor muscle (Figure 1(a)). In the second case, the brachioradial artery was well developed and branched off the second part of the axillary artery (posterior to the pectoralis minor muscle) (Figure 1(b)). In both cases, the brachioradial artery branched off the anterior aspect of the axillary artery and ran anterior to the roots of the median nerve.

**Figure 2:**
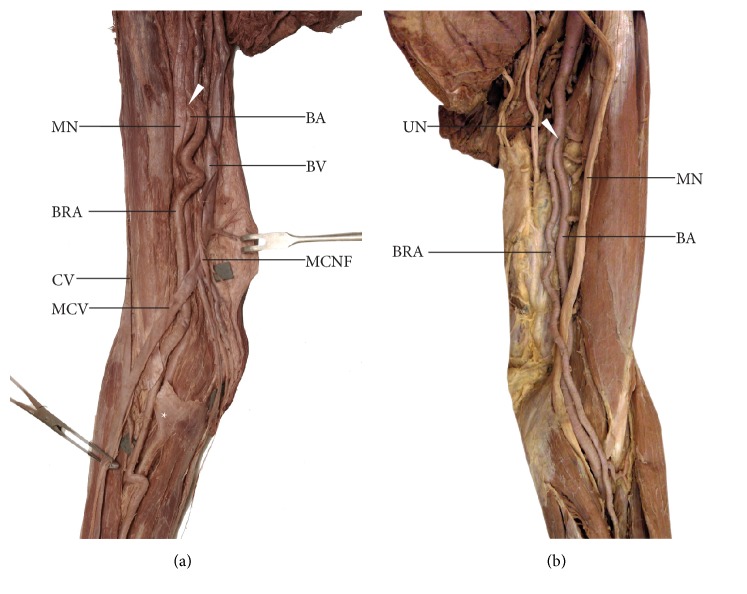
Two examples of the brachioradial artery originating in the arm. In both cases the brachioradial artery ran superficially to median nerve. (a) Right female upper limb. Anatomical relations within the medial bicipital groove. The brachioradial artery runs under the bicipital aponeurosis; within the forearm, it continues as the radial artery and occupies a typical position. (b) Left male upper limb. Two arterial trunks (brachioradial and “normal” brachial artery) are visible within the medial bicipital groove. In its course, the brachioradial artery crosses the anterior surface of the median nerve above the intercondylar line of the humerus running to the lateral side of this nerve.* BA*: brachial artery;* BV*: basilic vein;* BRA*: brachioradial artery;* CV*: cephalic vein;* MCNF*: medial cutaneous nerve of forearm;* MCV*: median cubital vein;* MN*: median nerve;* UN*: ulnar nerve.* White arrowhead* shows the origin of the brachioradial artery.

The nine remaining cases (7.5% of all limbs; 81.8% of brachioradial arteries) all arose on the arm within the medial bicipital groove (Figure 2). This variation was observed in four male and five female upper limbs. In this variant, the brachioradial artery branched off the medial aspect of the brachial artery and ran superficial to the median nerve, with its course initially being slightly medial to it (Figure 2). However, the brachial artery occupied a normal position behind the median nerve. The branching site of the brachioradial artery was located from 5 mm to 67 mm below the lower margin of the pectoralis major muscle. In eight specimens representing this variant, the brachioradial artery branched off below the profunda brachii artery. In one case, the brachioradial artery branched off at the level of the lower margin of the pectoralis major muscle, just above the origin of the profunda brachii artery. Further, along its course, the artery crossed the anterior surface of the median nerve between 57 mm and 95 mm from the intercondylar line of the humerus (mean 75 mm, ± 13 mm), moving to the lateral side of the nerve (Figure 2).

The brachioradial artery arose at a point between 126 mm and 260 mm above the intercondylar line (mean 178 mm, ± 44 mm). However, in typical cases the division of the brachial artery into terminal branches (namely, brachial and radial arteries) was located in the cubital fossa below the intercondylar line of humerus. The distance of the site of origin of the radial and brachioradial arteries from the intercondylar line of humerus is presented in Table 1. In all cases, the brachioradial artery ran under the deep (brachial and antebrachial) fascia and passed deep to the bicipital aponeurosis (Figure 2(a)).

### 3.3. Anatomical Variations of the Cubital Crossover and Radial Recurrent Arteries

Anastomosis between the brachioradial and “normal” brachial arteries in the cubital fossa was frequently observed (6/11 = 54.55%). This anastomosis, known as “cubital crossover” or “cubital connection”, was characterized by high variability. It occurred in three variants, classified as dominant, balanced, and minimal, depending on their diameter.

**Figure 3:**
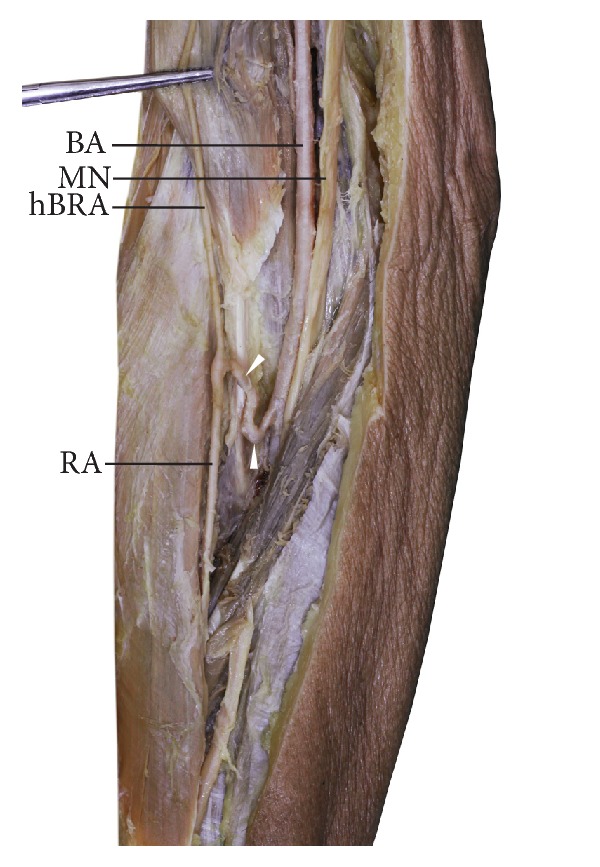
Dominant type of cubital crossover. Anterior view of the cubital fossa, right upper limb. This type of anastomosis between the brachioradial and “normal” brachial artery is characterized by a greater diameter than the brachial segment of the brachioradial artery. The brachioradial artery is present, but hypoplastic.* BA*: brachial artery;* hBRA*: hypoplastic brachial segment of the brachioradial artery;* MN*: median nerve;* RA*: radial artery.* White arrowheads* show the cubital crossover.

A dominant cubital crossover was found on one right male upper limb (1/11 = 9% of brachioradial artery cases; 1/120 = 0.83% of the examined upper limbs) (Figure 3). This anastomosis had a larger diameter than the brachial part (segment) of the brachioradial artery. In other words, brachioradial artery was present, but it was hypoplastic (Figure 3). In the described case, a hypoplastic brachioradial artery (1.85 mm in diameter), originating in the axillary fossa, joined a well-developed cubital crossover (3.21 mm in diameter) in the area of the cubital fossa and then continued in the forearm as the radial artery of a similar diameter. The dominant cubital crossover branched off the brachial artery (4.96 mm in diameter) 50 mm beneath the intercondylar line of the humerus, at the level of radial neck, from where it followed a tortuous course (Figure 3). The diameter of the brachioradial artery almost doubled (by 73.5%) just below the anastomosis. A single recurrent radial artery of 1.55 diameter arose 83 mm below the intercondylar line of the humerus.

**Figure 4:**
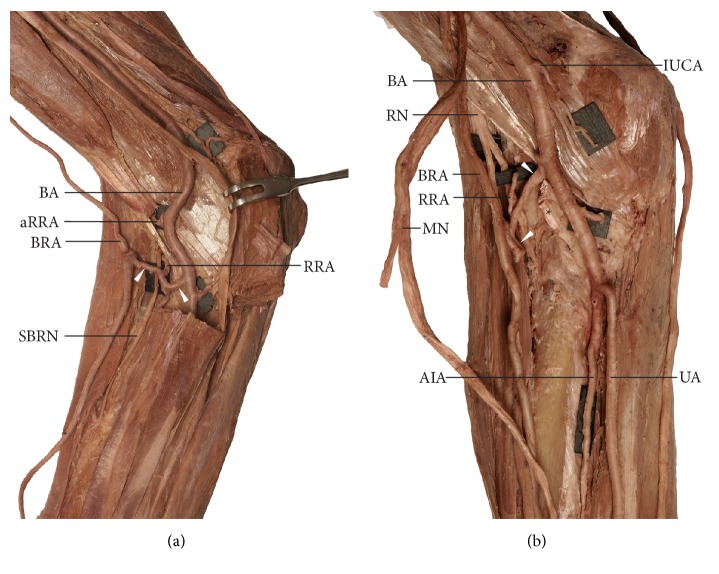
Balanced type of cubital crossover. This type of the anastomosis is characterized by the similar diameter to that of the brachioradial artery. (a) Anterior view of the cubital fossa, right male upper limb. The cubital crossover (marked by* white arrowheads*) runs anterior to the distal biceps tendon. (b) Anterior view of the cubital fossa, right female upper limb. In this case the cubital crossover (marked by* white arrowheads*) runs posterior to the distal biceps tendon.* AIA*: anterior interosseous artery;* aRRA*: accessory radial recurrent artery;* BA*: brachial artery;* BRA*: brachioradial artery;* IUCA*: inferior ulnar collateral artery;* MN*: median nerve;* RA*: radial artery;* RRA*: radial recurrent artery;* SBRN*: superficial branch of the radial nerve;* UA*: ulnar artery.

Three examples of balanced cubital crossover were observed (27.3% of brachioradial arteries; 2.5% of all examined limbs): two male limbs (1 right and 1 left) and one female limb (Figure 4). This anastomosis type was characterized by a diameter similar to that of the brachioradial artery: 2.45mm in male limbs and 2.34 mm in female limb. In this variant, the diameter of the brachioradial artery just below the anastomosis increased, respectively, by 17.1% (from 2.51 mm to 2.94 mm) in the male right limb, by 10.7% (from 2.71 mm to 3 mm) in the male left limb, and by 6% (from 2.33 mm to 2.47 mm) in the identified female limb. In all cases, small muscular branches ran downwards from the anastomosis, whereas a well-developed radial recurrent artery ran upwards, the diameter of which was 2.14 mm and 2.05 mm in the right and left male limbs and 1.35 mm in the female limb (Figure 4). In one case, in the right female limb, this anastomosis ran posterior to the distal biceps tendon (Figure 4(b)). This anastomosis was also characterized by a tortuous course. In all three cases, the presence of a recurrent radial artery was observed, which branched directly off this anastomosis. Moreover, an accessory recurrent radial artery was found in both male limbs, originating from the posteromedial aspect of the “normal” brachial artery (Figure 4(a)): 31 mm and 26 mm above the cubital crossover in the right and left male limbs. No accessory recurrent radial artery was observed in the female limb (Figure 4(b)).

**Figure 5:**
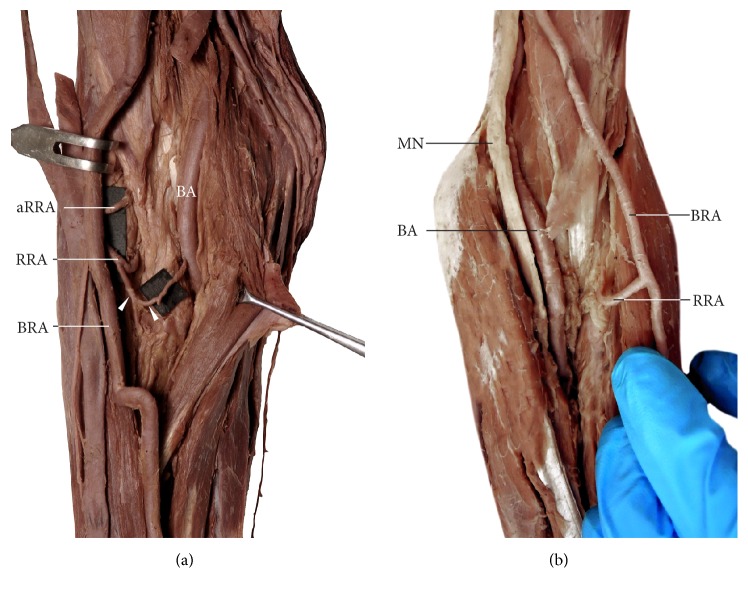
Two variants of the cubital crossover. (a) Minimal type of the cubital crossover (marked by* white arrowheads*). Anterior view of the cubital fossa, right female upper limb. (b) Absence of the cubital crossover. Anterior view of the cubital fossa, left female upper limb. In this case, two separate, unanastomosed arterial trunks ran within the cubital fossa.* aRRA*: accessory radial recurrent artery;* BA*: brachial artery;* BRA*: brachioradial artery;* MN*: median nerve;* RRA*: radial recurrent artery.

The minimal type of cubital crossover was observed in two cases (18.2% of brachioradial arteries; 1.7% of all tested limbs): one left male limb and one right female (Figure 5(a)). In this variant, no differences were found in the diameter of the brachioradial artery above and beneath the cubital crossover. However, in both cases, the diameter of the anastomosis decreased from 1.84 mm to 1.41 mm in the male upper limb and from 1.92 mm to 1.12 mm in the female upper limb just after the origin of the radial recurrent artery. The presence of an accessory recurrent radial artery was observed in one female limb (diameter 1.71 mm, 13 mm above the origin of the cubital crossover): the artery originated from the brachioradial artery (Figure 5(a)).

The cubital crossover was found to be absent in 45.4% of brachioradial arteries: in two male limbs (one right, one left) and in three female limbs (two left, one right) (Figure 5(b)). In these limbs, the radial recurrent artery originated from the brachioradial artery below the intercondylar line of the humerus (Figure 5(b)). An accessory recurrent radial artery was observed only in one of these cases (one female left limb). This artery originated from the “normal” brachial artery.

In summary, the variations related to the origin of the recurrent radial arteries observed in the examined upper limbs branched off the “normal” (typical) radial artery in 81.6% of cases (98/120), the posterior radioulnar division (called “trifurcation of the brachial artery”) in 9.2% (11/120), directly from the brachioradial artery in 5% (6/120), and the cubital crossover in 4.2% (5/120). The accessory radial artery was noted in 28 cases (28/120 = 23.3%), including four of the 11 limbs (36.4%) with a brachioradial artery.

### 3.4. Contribution of Radial or Brachioradial Arteries to Formation of the Superficial Palmar Arch

**Figure 6:**
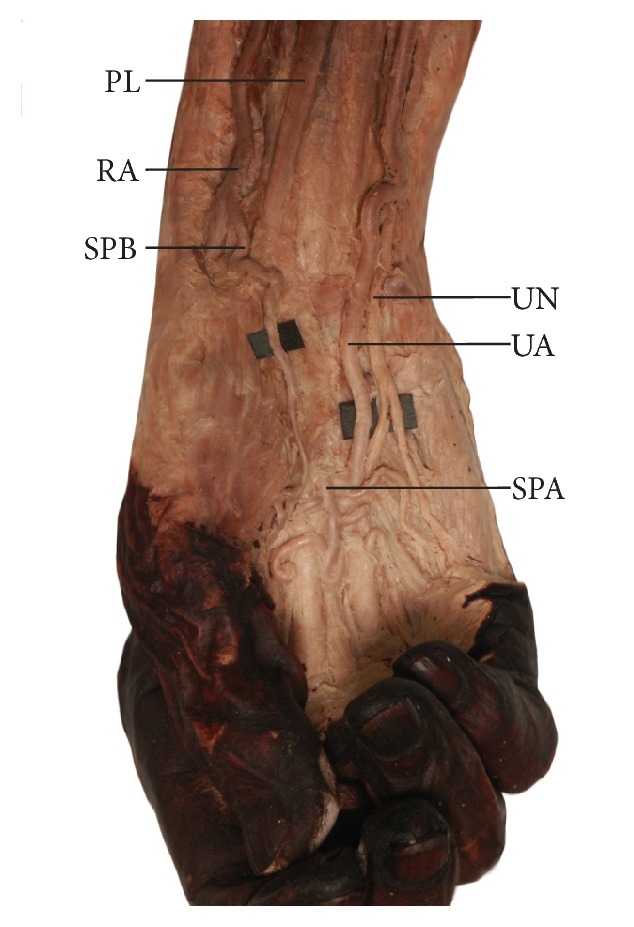
The most frequent variant of superficial palmar arch observed on the limbs showing a high origin to the radial artery (brachioradial artery). This type may be classified as a complete radioulnar arch in which a well-developed superficial palmar branch of radial (or, respectively, brachioradial) artery contributes to the radial half of the arch.* SPA*: superficial palmar arch;* SPB*: superficial palmar branch arising from the brachioradial artery;* PL*: tendon of palmaris longus muscle;* RA*: radial artery;* UA*: ulnar artery;* UN*: ulnar nerve.

Two basic types of superficial palmar arch were observed in the examined specimens. The first major type was a complete radioulnar arch, in which a well-developed superficial palmar branch of radial (or, respectively, brachioradial) artery contributed to the radial half of the arch (Figure 6). This type was revealed in eight of the upper limbs with a brachioradial artery present (8/11; 72.7%) and in 41 upper limbs (41/109; 37.6%) in which a “normal” radial artery was found. A significant difference was found between the limbs with a “typical” radial artery and those with a brachioradial artery with regard to the occurrence of a superficial palmar arch (p < 0.05). The second major type received its full contribution from the ulnar artery. This type was observed in three of the limbs with a brachioradial artery (3/11; 27.3%) and in 51 (51/109; 46.8%) in which a “normal” radial artery was found; the remaining limbs with a “normal” radial artery possessed rarer variants of an incomplete superficial palmar arch (17/109).  Table 2 compares the diameters of the radial, brachioradial, and ulnar arteries at the level of the wrist. No significant differences were found between the limbs with “normal” radial artery and the limbs with the brachioradial artery with regard to the formation of the deep palmar arch.

## 4. Discussion

### 4.1. Embryological Background

**Figure 7:**
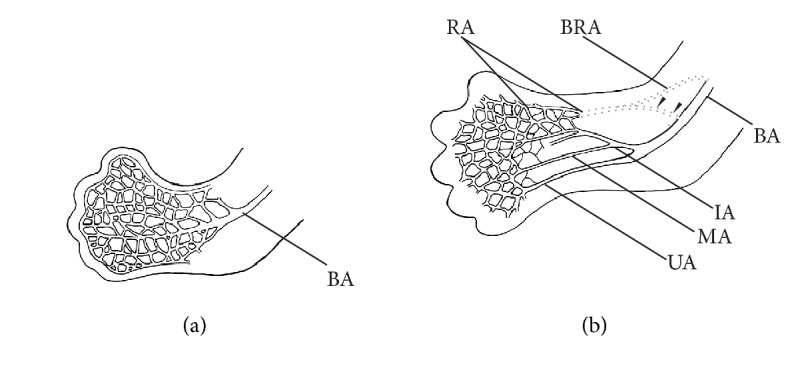
Schematic representation of the arterial remodeling in the developing upper limb between Carnegie stages 17 and 18. (a) Stage 17. Before this stage, the brachial artery branches into the capillary network allowing for the formation of different blood flow pathways. (b) Stage 18. The definitive origin of the radial artery is established at this stage. Also at this stage, the cubital crossover (*black arrowheads*) between the brachioradial and “normal” brachial artery may be formed in the place of typical origin of the radial artery (both typical origin and high origin of the radial artery have been marked by* dotted lines*).* BA*: brachial artery;* BRA*: brachioradial artery;* IA*: interosseous artery;* MA*: median artery;* RA*: radial artery;* UA*: ulnar artery. This figure is a modification of the drawing taken from Wysiadecki et al. (2017) under the terms of the Creative Commons Attribution 4.0 International License (https://creativecommons.org/licenses/by/4.0/), which permits unrestricted use, distribution, and reproduction in any medium.

Classical theories of development of upper limb arteries assumed that a gradual sprouting of arterial trunks takes place from a primitive axial artery [20, 38]. In turn, Rodríguez-Baeza et al. [22] propose a model based on the assumption that during normal morphogenesis, the upper limb arteries are formed by the union of superficial and deep pathways, implying that the superficial brachial artery is a consistent embryonic vessel. New models of the development of the upper limb arteries assume that the definitive arterial pattern of the upper limb is formed as a result of the isolation of main arterial trunks within the primitive capillary plexus [12, 13, 39–41]. The dominant vascular channels differentiate as a result of capillary remodeling (Figure 7). It can be assumed that such remodeling may give rise to anatomic variations in the arterial pattern, including the presence of atypical high origin of the radial artery.

**Figure 8:**
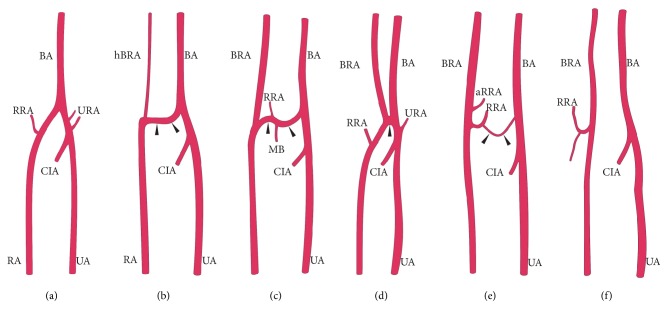
Selected anatomical variations of the cubital crossover (marked by* black arrowheads*) between the brachioradial and “normal” brachial artery. (a) Typical course of the radial artery. (b) Dominant type of cubital crossover with hypoplastic preanastomotic part of brachioradial artery. (c) Balanced type. (d) Arterial island (described by Piagkou et al. [42]). (e) Minimal type. (f) No anastomosis between the brachioradial and “normal” brachial artery within the cubital fossa.* aRRA*: accessory radial recurrent artery;* BA*: brachial artery;* BRA*: brachioradial artery;* CIA*: common interosseous artery;* hBRA*: hypoplastic preanastomotic part of brachioradial artery;* MB*: muscular branch;* RA*: radial artery;* RRA*: radial recurrent artery;* UA*: ulnar artery;* URA*: ulnar recurrent artery.

The cubital crossover may be considered as remnant of the connection between the primitive axial and superficial brachial arteries at the level of typical origin of the radial artery [12, 13, 15, 17, 22, 40, 41]. Different types of this anastomosis as well as numerous variations of radial and radial recurrent arteries can indicate various possible directions of rearrangements occurring at early stages of fetal development (Figures 7 and 8). In rare cases, two arteries (superficial brachial and brachial) may create an arterial complex (like an island pattern) at the level of the radial neck, which ends in a division into radial and ulnar arteries [42] (Figure 8(d)). Thus, an analysis of anatomical variations can contribute to a better understanding of the mechanisms that guide the morphogenesis of arteries [43]. Furthermore, many additional regulators, including genes, molecular signals, and hemodynamic forces, may play an important role in the formation of the definitive arterial pattern [40, 41, 44].

### 4.2. A Review of the Variations of the Brachioradial Artery

The prevalence of the high origin of the radial artery (brachioradial artery) according to different authors varies from 4.67% to 15.6% [7, 8, 10, 12, 14, 21, 22, 36, 37]. The frequency of the brachioradial artery given by selected authors is presented in Table 3. This variation was observed most often unilaterally [11–13, 21, 22, 45]. However, the identified dependence of the occurrence of the brachioradial artery in relation to gender and the side of the body varies between studies. In a study carried out on 750 upper limbs, McCormack et al. [10] found a radial artery with a high origin originating from the brachial artery in 57 right and 37 left upper limbs and from the axillary artery in 14 left and two right upper limbs. Rodríguez-Niedenführ et al. [12] noted this variant more frequently in women and on the right side. In the present study, no statistically significant differences were observed in the prevalence of the brachioradial artery with regard to gender or side of the body. However, the origin from the axillary artery was observed only in two right male upper limbs.

The brachioradial artery may take the origin from all parts of the axillary artery or, more commonly, it branches off the medial circumference of the brachial artery [7, 8, 10, 12–14, 21, 22, 36, 37, 45]. The frequency of the origin of brachioradial artery from the axillary or brachial artery recorded in the studies of other authors is presented in Table 3. According to Nasr [11], the mean distance between the intercondylar line of the humerus and the normal origin of RA was 38.7 ± 9.5 mm in male cadavers and 36.5 ± 8.5 mm in female cadavers. In the present study, the brachioradial artery usually arose between 126 mm and 260 mm above the intercondylar line of the humerus (mean 178 mm, ± 44 mm).

A cubital crossover is an anastomotic artery running in a variable manner between the brachioradial and “normal” brachial artery (Figures 8(b), 8(c), and 8(e)). Such “loop-like” formations were observed by Tiedemann [46], who was the first to systematically describe variations of the upper limb arteries and then by Adachi [7] in his classical studies. Docimo et al. [47] describe such anastomosis as “arterio-arterial malformation between a high origin radial artery and brachial artery” within the cubital fossa. In a study conducted by McCormack et al. [10], the cubital crossover was found in 17.8% of the limbs with a high origin of the radial artery, with only 5 cases (4.7% of limbs with the brachioradial artery; 26.3% of specimens with the anastomosis) in which a cubital anastomosis passed posterior to the distal biceps tendon. In turn, in a study of 384 upper limbs, Rodríguez-Niedenführ et al. [12] observed cubital anastomosis in 14 out of 53 limbs (26.4%) with brachioradial artery. The anastomosis adopted a “rectilinear form” in four cases (two in front and two behind the distal biceps tendon) and a “sling-like loop morphology” in 10 cases (six in front and four behind the distal biceps tendon). A very prominent anastomosis with slender preanastomotic part of the radial artery of high origin (Figure 8(b)) was observed by McCormack et al. [10] in 4.7% of limbs with the high origin of the radial artery, where 26.3% of specimens were with the anastomosis. A similar hypoplastic proximal (preanastomotic) segment of the brachioradial artery was described by von Haller as* vasa aberrantia *[48]. The present study distinguishes three types of cubital crossover (i.e., “cubital connection”) based on its diameter: dominant in one case (Figure 8(b)), balanced in three cases (Figure 8(c)), and minimal in two cases (Figure 8(e)). In our research, the cubital crossover was absent in five out of 11 (45,4%) upper limbs with brachioradial artery (Figure 8(f)).

Major anatomical variations of the upper limb arteries often coexist with the variations of radial recurrent arteries [18]. According to Vazquez et al. [18] the radial recurrent artery takes its origin most frequently from the radial artery (64,8%), posterior radioulnar division (9%), anterior radioulnar division (5.4%), brachioradial artery (7.8%), brachial artery (7.2%), ulno-interosseous trunk (2.7%), or even the interosseous trunk (0.3%). Our present findings indicate the recurrent radial artery branched off the “normal” (typical) radial artery in 81.6% of limbs, the posterior radioulnar division in 9.2%, directly from the brachioradial artery in 5%, and the cubital crossover in 4.2%. McCormack et al. [10] found the radial recurrent artery to arise from the cubital crossover in 10 out of 14 cases possessing this type of union. However, Rodríguez-Niedenführ et al. [12] found that when the brachioradial artery was present, the radial recurrent artery took origin from it in 46% of cases, from the “normal” brachial artery in 34%, and from the cubital crossover in 20%. In our study, when the brachioradial artery was present, the recurrent radial artery directly branched off the brachioradial artery in 54.6% of cases (6/11) or the cubital crossover in 45.4% of cases (5/11). Similar observations apply to those cases in which the accessory recurrent radial artery occurs. Vazquez et al. [18] found the accessory recurrent radial artery to be present in 103 (31%) out of 332 upper limbs, wherein the accessory recurrent radial artery always (100%) branched off the brachial artery, above the typical level of the origin of the radial artery, running posterior to the distal biceps tendon. Rodríguez-Niedenführ et al. [12] describe the occurrence of the accessory radial artery in 12 cases (22.6%) where the brachioradial artery was present. In the present study, the accessory radial artery was identified in four out of 11 cases (36.4%) where the brachioradial artery was also present.

### 4.3. Clinical Importance

Since the radial artery is often used in vascular, plastic, and reconstructive surgery [2, 3] and routinely used for puncture and cannulation [32, 33, 35], knowledge of its variations can be of great clinical significance. Transradial access can be hindered by the presence of an unusual origin and course of the vessel [26, 34]. It has been recently noted that the presence of a high origin of the radial artery (namely, the brachioradial artery) “considerably contributed to the development of tortuosity”, which can increase the risk of failure of transradial catheterization [49]. Anatomical variations of both radial and recurrent radial arteries may also influence the safety and success rate of plastic and reconstructive surgery. The presence of a recurrent radial artery may be useful in the radial forearm free flap surgeries. Hamahata et al. [16] stated that anastomosis using radial recurrent artery vessels is recommended as a strategy in free radial forearm transplantation for salvage operations. The superficial palmar branch of the radial artery can be also used in plastic and reconstructive surgery as the radial artery superficial palmar branch perforator flap [50–52]. Our present findings indicate that the prominent superficial palmar branch contributed to the formation of superficial palmar arch more often when the brachioradial artery occurred. The presence of atypical arterial patterns in the upper limb can be predicted based on the Color Doppler ultrasonography, which facilitate the assessment of the origin, course, variations, and locations of both arteries and accompanying veins [53, 54].

## 5. Conclusions

The brachioradial artery has several anatomical variations. Although it typically originates from the brachial artery, it can also, less frequently, originate from the axillary artery. An anastomosis can frequently be observed between the brachioradial and “normal” brachial arteries in the cubital fossa, known as a “cubital crossover” or “cubital connection”. A cubital crossover was observed in three variations classified as dominant, balanced, or minimal depending on the diameter. A complete radioulnar arch, in which a well-developed superficial palmar branch contributed to the radial half of the arch, was observed more often when the brachioradial artery was present as a variant. Orthopedic, hand, and plastic surgeons should be aware of anatomic variations of the radial artery both in planning and in conducting surgeries of the upper limb.

## Figures and Tables

**Table 1:** Comparison between the origin of the radial and brachioradial arteries in relation to the intercondylar line of the humerus.

**Typical origin of the radial artery, distance below the intercondylar line [mm]**					
	**Mean**	**Median**	**Minimal value**	**Maximal value**	**Standard deviation**
Male limbs	39.1	37.2	23.7	52.9	8.4

Female limbs	31.3	30	19.8	43.4	8.9

**Total**	**35.2**	**36.4**	**19.8**	**52.9**	**9.4**

**High origin of the radial artery (brachioradial artery), distance above the intercondylar line [mm]**					
	**Mean**	**Median**	**Minimal value**	**Maximal value**	**Standard deviation**

Male limbs	196	180	145	260	42

Female limbs	157	141	126	225	36

**Total**	**178**	**170**	**126**	**260**	**44**

**Table 2:** Comparison between the diameters of the ulnar and radial or brachioradial arteries at the level of the wrist.

**The diameters of the typical radial and ulnar arteries at the level of the wrist**										
	**Radial artery [mm]**					**Ulnar artery [mm]**				
	Mean	Median	Minimal value	Maximal value	Standard deviation	Mean	Median	Minimal value	Maximal value	Standard deviation
Male limbs	3.14	3.2	2.5	4.11	0.43	2.7	2.6	2.15	3.24	0.34

Female limbs	2.75	2.63	2.15	3.28	0.37	2.69	2.68	2.1	3.11	0.27

**Total**	**2.95**	**2.98**	**2.15**	**4.1**	**0.43**	**2.69**	**2.64**	**2.1**	**3.24**	**0.31**

**The diameters of the brachioradial and ulnar arteries at the level of the wrist**										
	**Brachioradial artery [mm]**					**Ulnar artery [mm]**				
	Mean	Median	Minimal value	Maximal value	Standard deviation	Mean	Median	Minimal value	Maximal value	Standard deviation

Male limbs	3.18	3.03	2.74	4.12	0.52	3.15	3.12	2,87	3.45	0.2

Female limb	3.02	3.08	2.02	3.65	0.57	2.85	2.86	2.03	3.43	0.49

**Total**	**3.1**	**3.03**	**2.02**	**4.12**	**0.53**	**3**	**3.08**	**2.03**	**3.45**	**0.39**

**Table 3:** Frequency of the brachioradial artery given by selected authors.

**Author, year of study**	**Sample (No. of limbs)**	**Frequency**		
		**Origin from axillary artery**	**Origin from brachial artery**	**All cases of the brachioradial artery**
Quain, 1844 [36]	429	30% of brachioradial arteries	70% of brachioradial arteries	12.4% of total amount of upper limbs

Adachi, 1928 [7]	410	2.2% (9/410) (31% of brachioradial arteries)	4.9% (20/410) (69% of brachioradial arteries)	7% of all limbs

McCormack et al., 1953 [10]	750	2.13% of total / 11.5 % of variations	12.14 % of total / 65.5 % of variations	14.27% of all specimens – 77% of all variations of upper limb arteries

Wathersby, 1956 [14]	408	-	-	15.6%

Keen, 1961 [21]	284	-	-	5.9%

Karlsson and Niechajev, 1982 [8]	82 patients with demonstrated anatomy of the whole upper extremity	1.22% of all patients (12.5% of brachioradial arteries)	8.54% of all patients (12.5% of brachioradial arteries)	97.5% of the cases in angiographic studies

Uglietta and Kadir, 1989 [37]	100 angiographic studies of the upper limb	1% of all cases / 12.5% of brachioradial arteries	7% of all cases / 87.5% of brachioradial arteries	8% of all examined cases

Rodríguez-Baeza et al., 1995 [22]	150	0.67% of total / 14.28% of brachioradial arteries	4% of total / 85.72% of brachioradial arteries	4.67%

Rodríguez-Niedenführ et al., 2001 [12]	384	3.1% of total (23% of brachioradial arteries)	3.1% of total (23% of brachioradial arteries)	13.8% of total amount of upper limbs and 20.3% out of 192 cadavers

Nasr, 2012 [11]	100	1% of total (1 out of 8 brachioradial arteries)	7% of total	8% of total amount of upper limbs

**Our present findings**	120	1.67% of all upper limbs and 18% of brachioradial arteries	7.5% limbs subjected to autopsy; 82% of brachioradial arteries	9.2% of total amount of upper limbs

## Data Availability

The data used to support the findings of this study are available from the corresponding author upon request.

## References

[1] Aragão J. A., da Silva A. C. F., Anunciação C. B., Reis F. P. (2017). Median artery of the forearm in human fetuses in northeastern Brazil: anatomical study and review of the literature. *Anatomical Science International*.

[2] Cikla U., Mukherjee D., Tumturk A., Baskaya M. K. (2018). Overcoming end-to-end vessel mismatch during superficial temporal artery–radial artery–M2 interposition grafting for cerebral ischemia: tapering technique. *World Neurosurgery*.

[3] Jennings W. C., Mallios A., Mushtaq N. (2017). Proximal radial artery arteriovenous fistula for hemodialysis vascular access. *Journal of Vascular Surgery*.

[4] Kachlik D., Hajek P., Konarik M., Krchov M., Baca V. (2016). Coincidence of superficial brachiomedian artery and bitendinous palmaris longus: a case report. *Surgical and Radiologic Anatomy*.

[5] Shetty S. D., Nayak B. S., Madhav N. V., Sirasanagandla S. R., Abhinitha P. (2012). The Abnormal Origin, course and the distribution of the arteries of the upper limb: a case report. *Journal for Clinical and Diagnostic Research*.

[6] Singer G., Marterer R., Till H., Schmidt B. (2018). A rare anatomic variation of the superficial palmar branch of the radial artery causing pain. *Surgical and Radiologic Anatomy*.

[7] Adachi B. (1928). *Das Arteriensystem der Japaner*.

[8] Karlsson S., Niechajev I. A. (1982). Arterial anatomy of the upper extremity. *Acta Radiologica*.

[9] Lee H., Moon Y., Park H. S., Kim H., Choi I. (2016). A radial artery originating from the thoracoacromial artery. *Surgical and Radiologic Anatomy*.

[10] McCormack L. J., Cauldwell E. W., Anson B. J. (1953). Brachial and antebrachial arterial patterns; a study of 750 extremities. *Surgery, Gynecology & Obstetrics*.

[11] Nasr A. Y. (2012). The radial artery and its variations: anatomical study and clinical implications. *Folia Morphologica*.

[12] Rodríguez-Niedenführ M., Vázquez T., Nearn L., Ferreira B., Parkin I., Sañudo J. R. (2001). Variations of the arterial pattern in the upper limb revisited: a morphological and statistical study, with a review of the literature. *Journal of Anatomy*.

[13] Rodríguez-Niedenführ M., Vázquez T., Parkin I. G., Sañudo J. R. (2003). Arterial patterns of the human upper limb: Update of anatomical variations and embryological development. *European Journal of Anatomy*.

[14] Weathersby H. T. (1956). Anomalies of brachial and antebrachial arteries of surgical significance. *Southern Medical Journal*.

[15] Wysiadecki G., Polguj M., Haładaj R., Topol M. (2017). Low origin of the radial artery: a case study including a review of literature and proposal of an embryological explanation. *Anatomical Science International*.

[16] Hamahata A., Nakazawa H., Takeuchi M., Sakurai H. (2012). Usefulness of radial recurrent artery in transplant of radial forearm flap: An anatomical and clinical study. *Journal of Reconstructive Microsurgery*.

[17] Rodríguez-Niedenführ M., Sañudo J. R., Vázquez T., Nearn L., Logan B., Parkin I. (2000). Anastomosis at the level of the elbow joint connecting the deep, or normal, brachial artery with major arterial variations of the upper limb. *Journal of Anatomy*.

[18] Vazquez T., Sañudo J. R., Carretero J., Parkin I., Rodríguez-Niedenführ M. (2013). Variations of the radial recurrent artery of clinical interest. *Surgical and Radiologic Anatomy*.

[19] Kaplanoglu H., Beton O. (2017). Evaluation of anatomy and variations of superficial palmar arch and upper extremity arteries with CT angiography. *Surgical and Radiologic Anatomy*.

[20] Standring S. (2008). Grays Anatomy: The Anatomical Basis of Clinical Practice. *Gray’s Anatomy: The Anatomical Basis of Clinical Practice*.

[21] Keen J. A. (1961). A study of the arterial variations in the limbs, with special reference to symmetry of vascular patterns. *American Journal of Anatomy*.

[22] Rodriguez-Baeza A., Nebot J., Ferreira B. (1995). An anatomical study and ontogenetic explanation of 23 cases with variations in the main pattern of the human brachio-antebrachial arteries. *Journal of Anatomy*.

[23] Funk G. F., Valentino J., McCulloch T. M., Graham S. M., Hoffman H. T. (1995). Anomalies of forearm vascular anatomy encountered during elevation of the radial forearm flap. *Head & Neck*.

[24] Venkataram A., Ellur S., Muninarayana D., Joseph V. (2016). Radial artery forearm flap anomaly: a rare anomaly and the importance of the proximal exploratory incision. *Journal of Hand and Microsurgery*.

[25] Vollala V. R., Nagabhooshana S., Bhat S. M. (2008). Trifurcation of brachial artery with variant course of radial artery: rare observation. *Anatomical Science International*.

[26] Zheng Y., Shao L., Mao J.-Y. (2014). Bilaterally symmetrical congenital absence of radial artery: A case report. *BMC Surgery*.

[27] Celik H. H., Sargon M. F., Konan A., Kural E. (1996). High brachial artery bifurcation: a report of 2 cases. *Bulletin De l'Association Des Anatomistes*.

[28] Nakatani T., Tanaka S., Mizukami S. (1997). Correspondence. Superficial brachial artery continuing as the common interosseous artery. *Journal of Anatomy*.

[29] Nkomozepi P., Xhakaza N., Swanepoel E. (2017). Superficial brachial artery: A possible cause for idiopathic median nerve entrapment neuropathy. *Folia Morphologica*.

[30] Fujii Y., Teragawa H., Soga J. (2013). Flow-mediated vasodilation and anatomical variation of the brachial artery (Double brachial artery) in healthy subjects and patients with cardiovascular disease. *Circulation Journal*.

[31] Gaudino M., Crea F., Cammertoni F., Mazza A., Toesca A., Massetti M. (2014). Technical issues in the use of the radial artery as a coronary artery bypass conduit. *The Annals of Thoracic Surgery*.

[32] Dharma S., Kedev S., Patel T., Rao S. V., Bertrand O. F., Gilchrist I. C. (2017). Radial artery diameter does not correlate with body mass index: A duplex ultrasound analysis of 1706 patients undergoing trans-radial catheterization at three experienced radial centers. *International Journal of Cardiology*.

[33] Li X., Fang G., Yang D. (2016). Ultrasonic technology improves radial artery puncture and cannulation in intensive care unit (ICU) shock patients. *Medical Science Monitor*.

[34] Patel T., Shah S., Pancholy S., Rao S., Bertrand O. F., Kwan T. (2014). Balloon-assisted tracking: a must-know technique to overcome difficult anatomy during transradial approach. *Catheterization and Cardiovascular Interventions*.

[35] Wessel E., Hessel K., Glaros A., Olinger A. (2015). Quantification of the distal radial artery for improved vascular access. *Folia Morphologica*.

[36] Quain R. (1844). *The Anatomy of The Arteries of The Human Body: and Its Application to Pathology and Operative Surgery, with A Series of Lithographic Drawings*.

[37] Uglietta J. P., Kadir S. (1989). Arteriographic study of variant arterial anatomy of the upper extremities. *CardioVascular and Interventional Radiology*.

[38] Singer E. (1933). Embryological pattern persisting in the arteries of the arm. *The Anatomical Record*.

[39] Lin F.-J., Tsai M.-J., Tsai S. Y. (2007). Artery and vein formation: A tug of war between different forces. *EMBO Reports*.

[40] Vargesson N. (2003). Vascularization of the developing chick limb bud: Role of The TGF*β* signalling pathway. *Journal of Anatomy*.

[41] Rodríguez-Niedenführ M., Burton G. J., Deu J., Sañudo J. R. (2001). Development of the arterial pattern in the upper limb of staged human embryos: Normal development and anatomic variations. *Journal of Anatomy*.

[42] Piagkou M., Totlis T., Panagiotopoulos N.-A., Natsis K. (2016). An arterial island pattern of the axillary and brachial arteries: a case report with clinical implications. *Surgical and Radiologic Anatomy*.

[43] Bergman R. A. (2011). Thoughts on human variations. *Clinical Anatomy*.

[44] Carmeliet P. (2003). Blood vessels and nerves: Common signals, pathways and diseases. *Nature Reviews Genetics*.

[45] Yagain V. K., Dave M. R., Anadkat S. (2012). Unilateral high origin of radial artery from axillary artery. *Folia Morphologica*.

[46] Tiedemann F. (1822). *Friderici Tiedemann Tabulae Arteriarum Corporis Humani/Friederich Tiedemann’s Abbildungen der Pulsadern des menschlichen Koerpers*.

[47] Docimo S., Kornitsky D. E., Hill R. V., Elkowitz D. E. (2009). Arterio-arterial malformation between a high origin radial artery and brachial artery within the cubital fossa - Its clinical and embryological significance: A case report. *Cases Journal*.

[48] von Haller A. (1753). *Iconum Anatomicarum Quibus Praecipuae Partes Corporis Humani Exquisita Cura Delineatae Continentur Fasciculus I-IV*.

[49] Ostojić Z., Bulum J., Ernst A., Strozzi M., Marić-Bešić K. (2015). Frequency of radial artery anatomic variations in patients undergoing transradial heart catheterization. *Acta clinica Croatica*.

[50] Chi Z., Yang P., Song D. (2017). Reconstruction of totally degloved fingers: a novel application of the bilobed spiraled innervated radial artery superficial palmar branch perforator flap design provides for primary donor-site closure. *Surgical and Radiologic Anatomy*.

[51] Chi Z., Pafitanis G., Pont L. E. (2017). The use of innervated radial artery superficial palmar branch perforator free flap for complex digital injuries reconstruction. *Journal of Plastic Surgery and Hand Surgery*.

[52] Zekavica A., Milisavljević M., Erić D. (2017). Vascular anatomy of the thenar eminence: Its relevance to a pedicled or free thenar flap. *Folia Morphologica*.

[53] Chen L., Chen J., Hu B., Jiang L.-X. (2017). Sonographic findings of the bifid median nerve and persistent median artery in carpal tunnel: A preliminary study in Chinese individuals. *Clinics*.

[54] Sun R., Ding Y., Sun C. (2016). Color doppler sonographic and cadaveric study of the arterial vascularity of the lateral upper arm flap. *Journal of Ultrasound in Medicine*.

